# Parameter estimates for trends and patterns of excess mortality among persons on antiretroviral therapy in high-income European settings

**DOI:** 10.1097/QAD.0000000000002387

**Published:** 2019-11-11

**Authors:** Adam Trickey, Ard van Sighem, John Stover, Sophie Abgrall, Sophie Grabar, Fabrice Bonnet, Juan Berenguer, Christoph Wyen, Jordi Casabona, Antonella d’Arminio Monforte, Matthias Cavassini, Julia del Amo, Robert Zangerle, M. John Gill, Niels Obel, Jonathan A.C. Sterne, Margaret T. May

**Affiliations:** a Population Health Sciences, University of Bristol, Bristol, UK; b Stichting HIV Monitoring, Amsterdam, The Netherlands; c Avenir Health, Glastonbury, Connecticut, USA; d Department of Internal Medicine, Antoine Béclère Hospital, Clamart; e University of Paris Saclay, Paris-Sud University, UVSQ; f CESP INSERM U1018, Le Kremlin-Bicêtre; g Sorbonne Université, INSERM, Institut Pierre Louis d’épidemiologie et de Santé Publique (IPLESP), Unité de Biostatistique et d’épidémiologie Groupe hospitalier Cochin Broca Hôtel-Dieu, Assistance Publique Hôpitaux de Paris (AP-HP), and Université Paris Descartes, Sorbonne Paris Cité, Paris; h University of Bordeaux, ISPED, INSERM U1219 and CHU de Bordeaux, Bordeaux, France; i Hospital General Universitario Gregorio Marañón, Instituto de Investigación Sanitaria Gregorio Marañón (IiSGM), Madrid, Spain; j First Department of Internal Medicine, University of Cologne, Cologne, Germany; k CEEISCAT/Agència de Salut Pública de Catalunya, Campus Can Ruti and CIBERESP, Badalona, Catalonia, Spain; l Clinic of Infectious and Tropical Diseases, Department of Health Sciences, ASST Santi Paolo e Carlo, University of Milan, Milan, Italy; m Service of Infectious Diseases, Lausanne University Hospital and University of Lausanne, Lausanne, Switzerland; n National Epidemiology Center, Carlos III Health Institute, Madrid, Spain and National Plan on AIDS, Ministry of Health, Madrid, Spain; o Innsbruck Medical University, Austria; p Division of Infectious Diseases, University of Calgary, Calgary, Canada; q Department of Infectious Diseases, Copenhagen University Hospital, Rigshospitalet, Denmark.

**Keywords:** AIDS, cause-specific, cohort, death, duration, HIV, United Nations Programme on HIV/AIDS

## Abstract

**Introduction:**

HIV cohort data from high-income European countries were compared with the UNAIDS Spectrum modelling parameters for these same countries to validate mortality rates and excess mortality estimates for people living with HIV (PLHIV) on antiretroviral therapy (ART).

**Methods:**

Data from 2000 to 2015 were analysed from the Antiretroviral Therapy Cohort Collaboration (ART-CC) for Austria, Denmark, France, Italy, the Netherlands, Spain, and Switzerland. Flexible parametric models were used to compare all-cause mortality rates in the ART-CC and Spectrum. The percentage of AIDS-related deaths and excess mortality (both are the same within Spectrum) were compared, with excess mortality defined as that in excess of the general population mortality.

**Results:**

Analyses included 94 026 PLHIV with 585 784 person-years of follow-up, from which there were 5515 deaths. All-cause annual mortality rates in Spectrum for 2000–2003 were 0.0121, reducing to 0.0078 in 2012–2015, whilst the ART-CC's corresponding annual mortality rates were 0.0151 [95% confidence interval (95% CI): 0.0130–0.0171] reducing to 0.0049 (95% CI: 0.0039–0.0060). The percentage of AIDS-related deaths in Spectrum was 74.7% in 2000–2003, dropping to 43.6% in 2012–2015. In the ART-CC, AIDS-related mortality constitutes 45.3% (95% CI: 38.4–52.9%) of mortality in 2000–2003 and 26.7% (95% CI: 19–46%) between 2012 and 2015. Excess mortality in the ART-CC was broadly similar to the Spectrum estimates, dropping from 75.3% (95% CI: 60.3–95.2%) in 2000–2003 to 30.7% (95% CI: 25.5–63.7%) in 2012–2015.

**Conclusion:**

All-cause mortality assumptions for PLHIV on ART in high-income European settings should be adjusted in Spectrum to be higher in 2000–2003 and decline more quickly to levels currently captured for recent years.

## Abstract 2

Supplemental Digital Content is available in the text

## Introduction

Mortality rates among people living with HIV (PLHIV) taking antiretroviral therapy (ART) have declined since the millennium in high-income European countries [1,2] because of increasingly effective ART regimens that are easy to take and have minimal side effects, as well as other improvements in HIV care [3]. Changes to HIV treatment guidelines, which now recommend that PLHIV start ART as soon as possible after diagnosis regardless of their CD4 count, will also have impacted mortality in recent years [4]. Many studies have shown higher mortality during the first year of ART than subsequently [5–7], but this early mortality has declined in recent years because fewer PLHIV start ART with severe immunodeficiency [1]. Causes of death among PLHIV on ART have also changed, with a smaller proportion of AIDS-related deaths [2]. As the HIV-positive population ages, the proportion of deaths from causes associated with ageing, such as cancer and cardiovascular disease, has increased [2,8].

Spectrum (www.unaids.org/en/dataanalysis/datatools/spectrum-epp) is a comprehensive set of models of the HIV epidemics in countries around the world that is used by the United Nations Programme on HIV/AIDS (UNAIDS) to produce country-specific estimates of all-cause and AIDS-related mortality in PLHIV [9]. Spectrum model outputs are used by governments, researchers, and policy-makers globally, so it is important that these are as accurate as possible. To test the accuracy of the Spectrum mortality estimates, UNAIDS sought to compare methods, parameters, and outputs of the Spectrum models with those of observational HIV cohorts with good cause of death classifications.

We used data from the Antiretroviral Therapy Cohort Collaboration (ART-CC) [10] of clinical HIV cohorts in seven high-income European countries to validate Spectrum model parameters and outputs. Analyses compared excess mortality estimates for PLHIV on ART in ART-CC with those from Spectrum. The secondary aims were to investigate the association between duration of ART and mortality; compare mortality rates by calendar period in the ART-CC with trends estimated using Spectrum; compare the proportion of deaths on ART that are because of AIDS in the ART-CC with Spectrum; and provide updated mortality rates to parameterize Spectrum for high-income European countries and enable more accurate estimates of excess deaths because of AIDS.

## Methods

### Antiretroviral Therapy Cohort Collaboration data

The ART-CC combines data from HIV cohorts in Europe and North America. Eligible patients are HIV-1 positive, aged at least 16 years, and started ART on at least three drugs without having previously taken antiretrovirals. All contributing cohorts have been approved to use their data for research by institutional review boards or ethics committees. The cohorts use standardized methods of data collection, with follow-up visits scheduled at least every 6 months. The dataset is described in detail elsewhere (www.bristol.ac.uk/art-cc/) [10]. Only European ART-CC cohorts were included in this study as the North American cohorts within NA-ACCORD are more representative of the US and Canadian populations of PLHIV than those in the ART-CC [11]. The European cohorts included in this study were those representative of national populations of PLHIV in their countries because of their nationwide geographical coverage and because they contain a high percentage of the PLHIV on ART in that country, for example, AHIVCOS contains over 85% of Austria's PLHIV on ART [12]. The countries and cohorts analysed were Austria (AHIVCOS), Denmark (DHK), France (ANRS C03 Aquitaine cohort, ANRS CO4 French Hospital Database on HIV FHDH), Italy (ICONA), Netherlands (ATHENA), Spain (Co-RIS, PISCIS, VACH), and Switzerland (SHCS). The ART-CC 2015 data update was used (ART-CC receives data from its cohorts in cycles, e.g. the previous update was in 2013). The latest ART start date in this analysis was 9 January 2015 and the latest follow-up date used was 31 December 2015.

Follow-up started when PLHIV started ART and ended at the earliest of death, loss to follow-up (LTFU), or administrative censoring (a cohort-specific database closing date). If a patient's last clinical observation was more than a year before the cohort-specific database close date and they were not known to have died, then they were considered lost to follow-up. Alternative definitions of LTFU using gaps of no contact of 1.5 and 2 years were investigated. Data on follow-up between 2000 and 2015 were analysed, for comparability with Spectrum.

### Coding causes of death

Data on causes of death were obtained through either linkage with Vital Statistics agencies and hospitals or through active follow-up and physician report, depending on the cohort. An adapted version of the Cause of Death (CoDe) project protocol (www.cphiv.dk/CoDe.aspx) was used to classify causes of death [13]. When ICD-9 or ICD-10 codes or free-text information were available, causes of death were classified by a computer algorithm and a clinician. When either ICD-9 or ICD-10 codes or free-text information were unavailable, then each death was independently classified by two clinicians. Panel discussion was used to resolve disagreements between clinicians and/or the algorithm. Deaths were classified as AIDS-related if a serious AIDS-defining condition had been recorded a year prior to the death, and/or a low CD4^+^ count (<100 cells/μl) was the last recorded CD4^+^ count prior to death (within a year if on treatment, 18 months if off treatment), and there was a diagnosis compatible with AIDS as a cause of death.

### Spectrum data

European country-specific Spectrum outputs were made available to us by those with access to the data for each year from 2000 onwards. The outputs used were the number of PLHIV on ART, the number of AIDS deaths among persons on ART, and the number of non-AIDS deaths among persons on ART (estimated based on non-AIDS mortality rates published in the 2017 World Population Prospects data). Mortality rates in Spectrum's 2018 round of estimates were based on a 2013 analysis of Collaboration of Observational HIV Epidemiological Research in Europe (COHERE) data following methods used by the International epidemiologic Databases to Evaluate AIDS (IeDEA) collaboration to estimate mortality rates of PLHIV on ART in low-income and middle-income countries [14]. Mortality rate trends in Spectrum for Western Europe were calculated by fitting incidence curves to program data on new diagnoses and vital registration data on AIDS deaths. These previous Spectrum estimates did not take into account time trends in on-ART mortality.

This study aimed to compare default assumptions and parameters in Spectrum with available cohort data. Therefore, the Spectrum data were generated removing customized adjustments to the Spectrum default rates – modifications in the on-ART mortality rates to better match program reports of AIDS deaths. Removing these customized adjustments and using the default patterns meant the Spectrum outputs presented in this analysis were different from the published UNAIDS global estimates for Europe [15]. For comparability with the ART-CC, the annual data were grouped into the years 2000–2003, 2004–2007, 2008–2011, and 2012–2015. Using the Spectrum data, all-cause mortality rates, AIDS-related mortality rates (calculated as the excess mortality above that of the age-matched and sex-matched general population), and the percentage of deaths that were AIDS-related were estimated overall and by country. Annual mortality rates were calculated by dividing the number of deaths by the number of PLHIV alive at the midpoint of that year, presented as rates per person.

LTFU in Spectrum only affects the distribution of ART patients by time on ART as the input to Spectrum is the number of PLHIV on ART. High rates of LTFU mean that more people need to start ART to match the program reports of numbers on ART. As on-ART mortality is higher in the first year of treatment, higher LTFU rates are associated with higher overall ART-related mortality.

### Analyses

ART-CC data were reshaped to separate each calendar year of follow-up for each individual person. Age (grouped as 16–24, 25–34, 35–44, and ≥45 years) was updated for each calendar year of follow-up for each person. The duration on ART for each person was updated for each calendar year and categorized firstly as 0–6, 7–11, and at least 12 months, then secondly as 0 to less than 1, 1 to less than 2, 2 to less than 3, 3 to less than 4, and at least 4 years, to see which categorization best fitted the data. Categories were chosen to match those used in Spectrum.

For the analyses on duration of ART using data from the ART-CC, combined European rates of and incidence rate ratios (IRRs) for all-cause mortality were estimated using mixed-effects Poisson models with cohort as the panel variable. These models were adjusted for calendar year, sex, age, injecting drug use (IDU) transmission route, CD4^+^ at ART start (categorized as 0–49, 50–99, 100–199, 200–249, 250–349, 350–499, and ≥500 cells/μl), and duration of ART (using both sets of duration categories described above). There were no missing data for any of these variables.

For the analyses comparing country-specific mortality rates by calendar year group, the Spectrum rates were compared with crude and selected rates from ART-CC. Crude rates were stratified by calendar year group, and rates were estimated for a non-IDU men, aged 35–44, with a CD4^+^ count at ART start of 100–199 cells/μl, who had been on ART for 1–2 years – referred to henceforth as ‘selected rates’. We used flexible parametric survival models on the odds scale with one degree of freedom, equivalent to a log-logistic survival model [16]. For all Europe-wide comparisons between the ART-CC and Spectrum, the country-specific ART-CC mortality rates were weighted proportional to the number of PLHIV in each country in Spectrum's data. To investigate if mortality rates changed differently over calendar year group according to CD4^+^ count at ART start, a Poisson model was fit with an interaction term between CD4^+^ cell count group (categorized as above) and calendar year group, adjusting for variables as above.

AIDS-related mortality rates were compared between ART-CC and Spectrum. In Spectrum, AIDS-related mortality is calculated as any mortality among PLHIV that exceeds that in the age-matched and sex-matched general population, with mortality rates taken from the UN Department of Economic and Social Affairs (www.un.org/development/desa/en/). In the ART-CC, we calculated AIDS-related mortality using both cause-specific mortality and excess mortality (for a more direct comparison with Spectrum estimates). Flexible parametric models were used, adjusting only for calendar year group. Non-AIDS-related mortality (including deaths coded as unknown/unclassifiable) was censored in these analyses. In sensitivity analyses, we used a range of assumptions about the unknown/unclassifiable causes of deaths to estimate upper and lower bounds and a middle estimate for the proportion of deaths that were AIDS-related. These were that the proportion of AIDS-related deaths among those coded as unknown/unclassifiable was zero; equal to the proportion of coded deaths that were AIDS-related (sampling from a binomial distribution) – the middle estimate; and one. Coding all unknown/unclassifiable deaths as AIDS-related will overestimate the number of AIDS-related deaths because the available information for some of these deaths strongly suggested that they were not from AIDS, although it was not sufficient to identify a classifiable cause of death.

Excess mortality was calculated relative to the United Nation's sex-matched and age-matched general population mortality rates. The percentage of excess deaths in ART-CC was calculated by dividing the excess mortality rate by the overall mortality rate, with Bayesian 95% credibility intervals accounting for parameter uncertainty calculated using Winbugs 1.4.3. All other analyses used Stata version 15.1.

## Results

The ART-CC dataset contained 94026 PLHIV: 25 534 (27.2%) women and 9357 (10%) with IDU transmission. During 585 784 person-years of follow-up after 2000, there were 5515 deaths. At the time of starting ART, median age was 37 years (IQR 31–45), median CD4^+^ count was 252 cells/μl (IQR 126–377), median HIV-1 viral load was 63 160 (IQR 11 900–199 800) copies/ml and 17 127 (18.2%) PLHIV had been diagnosed with AIDS. When LTFU was defined as no clinical follow-up a year before the administrative censoring date, 20 763 (22.1%) PLHIV were considered lost, reducing to 15513 (16.5%) and 6850 (7.3%) for gaps of 1.5 and 2 years, respectively.

### Antiretroviral therapy duration

**Fig. 1:**
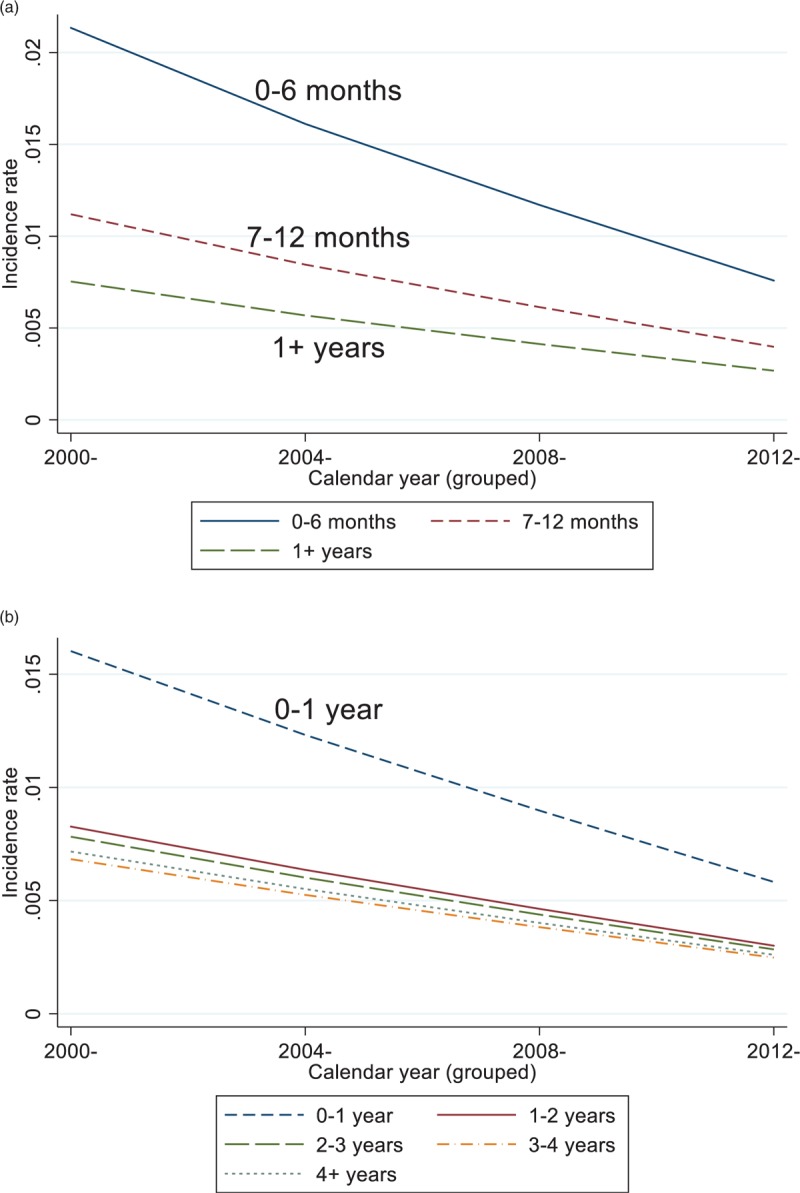
Antiretroviral Therapy Cohort Collaboration trends across calendar years in mortality rates by duration of antiretroviral therapy for (a) 0–6, 7–12 months, and at least 1 year; and (b) 0–1, 1–2, 2–3, 3–4, and at least 4 years.

^∗^Presented for a man with noninjecting drug use transmission, aged 35–44 years, with baseline CD4^+^ of 250–349 cells/μl.

When duration of ART was categorized as 0–6, 7–11, and at least 12 months, mortality rates were the highest during the first 0–6 months on ART (Fig. 1a and Supplementary Table 1). For a man with non-IDU transmission, aged 16–24 years, and baseline CD4^+^ count 100–199 cells/μl in their first 0–6 months on ART, the annual mortality rate in 2000–2003 was 0.017 (95% CI 0.011–0.022) per person, declining to 0.006 (0.004, 0.008) between 2012 and 2015. The adjusted mortality IRRs comparing 7–12 and at least 12 with 0–6 months ART duration were 0.52 (95% CI 0.47–0.59) and 0.35 (0.33–0.38), respectively. When duration was categorized as 0–1, 1–2, 2–3, 3–4, and at least 4 years, mortality rates were highest in the first year of ART (Fig. 1b and Supplementary Table 1). For a non-IDU man, aged 16–24 years with baseline CD4^+^ count 100–199 cells/μl in their first year on ART, the 2000–2003 annual mortality rate was 0.013 (0.009–0.017), declining to 0.005 (0.003–0.006) in 2012–2015. The adjusted IRRs compared with the first year of ART were: 0.52 (95% CI 0.47–0.57) for 1–2 years, 0.49 (0.44–0.54) for 2–3 years, 0.43 (0.38–0.47) for 3–4 years, and 0.44 (0.41, 0.48) for at least 4 years on ART.

### Calendar year period

**Fig. 2:**
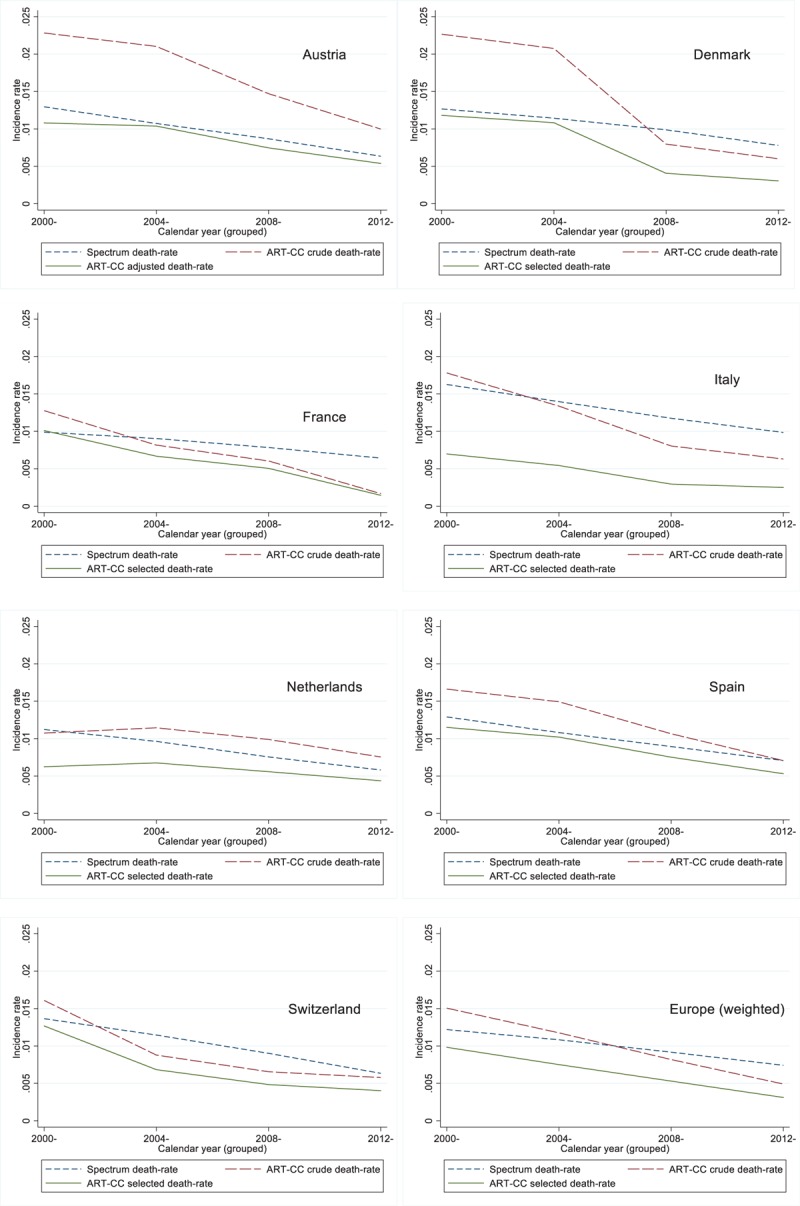
Comparison of Antiretroviral Therapy Cohort Collaboration mortality rates (crude and selected^∗^) against Spectrum model inputs.

^∗^A noninjecting drug use man, aged 35–44 years, with a CD4^+^ count at antiretroviral therapy start of 100–199 cells/μl, who had been on antiretroviral therapy for 1–2 years.

Figure 2 shows crude and selected country-specific comparisons of mortality rates between the ART-CC and Spectrum estimates, by calendar year period: Supplementary Table 2 provides further detail. For Europe overall and all the countries analysed except Austria and the Netherlands, the ART-CC crude mortality rates were higher than the Spectrum estimates in the 2000–2003 period but lower by 2012–2015. This indicates that the Spectrum mortality rates are not declining as quickly as those in the ART-CC. France and Switzerland had the lowest mortality rates in ART-CC data, whilst Austria and Denmark had the highest. The ART-CC analyses suggested that in some countries declines in mortality were less rapid in more recent years: this was not apparent in the Spectrum modelling. Mortality rates appeared to decline sooner in France and Switzerland than in the other countries analysed. Using cause of death coding, estimated rates of AIDS-related mortality, in ART-CC were 0.0049 in 2000–2003, 0.0028 in 2004–2007, 0.0020 in 2008–2011, and 0.0007 in 2012–2015: much lower than in Spectrum (0.0091, 0.0074, 0.0054, and 0.0032 in the same periods). However, estimated excess mortality rates in ART-CC were more similar to those in Spectrum (0.0115, 0.0082, 0.0045, and 0.0021 in the same periods), although excess mortality declined more across calendar periods in ART-CC than in Spectrum. Supplementary Table 3 shows that declines in mortality rates over calendar years were less pronounced among PLHIV starting ART with higher than lower CD4^+^ counts.

### Causes of death

**Fig. 3:**
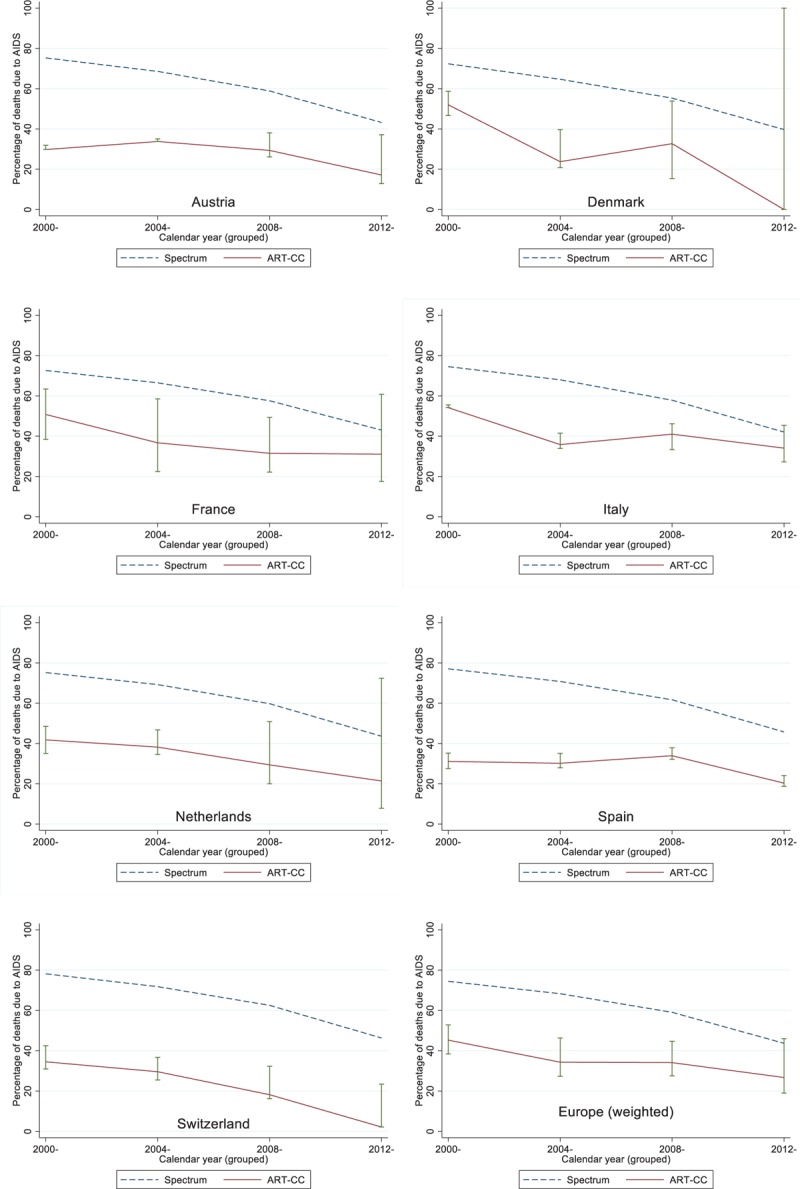
Comparisons of the percentage of deaths because of AIDS in the Antiretroviral Therapy Cohort Collaboration against Spectrum model inputs, for separate European countries.

Table 1 shows the distribution of specific causes of death in the ART-CC by calendar year period. The percentage of deaths in ART-CC that were classified as AIDS-related declined from 36% in 2000–2003 to 27% in 2004–2007, 25% in 2008–2011, and 13% in 2012–2015. However, the percentage classified as unknown increased from 16% in 2000–2003 to 19% in 2004–2007, 20% in 2008–2011, and 39% in 2012–2015. Supplementary Table 4 shows lower and upper bounds, and middle estimate of the percentage of AIDS-related deaths, based on different assumptions about the unclassified/unknown deaths. Figure 3 shows a comparison of the percentage of deaths because of AIDS between Spectrum and the ART-CC. Supplementary Table 5 gives country-specific estimates and confidence intervals. The estimated percentage of AIDS deaths was lower in ART-CC than in the Spectrum estimates for all calendar year periods, even after assuming that all unknown/unclassifiable deaths were because of AIDS. For 2000–2011, the percentage of AIDS deaths was lower in ART-CC than in the Spectrum estimates for each country analysed, even after assuming that all unknown/unclassifiable deaths in ART-CC were because of AIDS. For 2012–2015, the percentage of AIDS deaths was lower in ART-CC than in the Spectrum estimates for each country, but this was no longer the case for Denmark, France, Italy, and the Netherlands when assuming all unknown/unclassifiable deaths in ART-CC were AIDS-related. Estimated percentage excess mortality in ART-CC (75.3% in 2000–2003, 66.4% in 2004–2007, 52.4% in 2008–2011, 30.7% in 2012–2015) was similar to Spectrum (74.7, 68.4, 59.1, and 43.6% in the same calendar periods) except for 2012–2015.

**Table 1:** Distribution of cause-of-death in the Antiretroviral Therapy Cohort Collaboration by calendar year period.

	Calendar year period			
Cause of death	2000–2003	2004–2007	2008–2011	2012–2015
AIDS	496 (36%)	454 (27%)	429 (25%)	90 (13%)
Cardiovascular	52 (4%)	94 (6%)	85 (5%)	30 (4%)
Liver	98 (7%)	111 (7%)	139 (8%)	50 (7%)
Non-AIDS infection	64 (5%)	107 (6%)	84 (5%)	18 (3%)
Cancer (non-AIDS or liver)	128 (9%)	218 (13%)	281 (16%)	103 (15%)
Other	200 (14%)	242 (14%)	219 (13%)	83 (12%)
Respiratory	14 (1%)	28 (2%)	30 (2%)	26 (4%)
Substance abuse	47 (3%)	37 (2%)	50 (3%)	10 (1%)
Unknown/unclassifiable	225 (16%)	331 (19%)	337 (20%)	274 (39%)
Unnatural	56 (4%)	78 (5%)	70 (4%)	26 (4%)
Total	1380 (100%)	1700 (100%)	1725 (100%)	710 (100%)

## Discussion

Overall, mortality rates in Spectrum appeared to have been under-estimated for the early years of ART and over-estimated for the later years, compared with the ART-CC data. Mortality rates were much higher for PLHIV in the ART-CC during their first 0–6 months or first year on ART than afterwards. Higher mortality rates at the beginning of ART are likely because of PLHIV presenting late for treatment with compromised immune systems. Correspondingly, the decline in mortality over calendar years is less pronounced for PLHIV presenting with higher CD4^+^ counts. In both the ART-CC and Spectrum, the percentage of deaths because of AIDS decreased over the calendar years. The percentage of deaths because of AIDS appeared to be over-estimated in Spectrum because it was higher than in the ART-CC, even when assuming all unknown/unclassifiable deaths in the ART-CC were because of AIDS. However, the percentage of deaths among PLHIV in the ART-CC that were in excess of the general population mortality was broadly similar to that in Spectrum.

These comparisons between Spectrum and the ART-CC draw attention to how excess mortality is estimated among PLHIV on ART in high-income settings. Extra mortality can manifest as deaths because of AIDS but can also be because of other causes that are more common among PLHIV than in the general population, such as increased inflammation, hepatitis C virus coinfection, smoking, and IDU [17–21]. Comparing mortality rates between Spectrum and the ART-CC indicates that Spectrum's assumptions need to be adjusted to reflect higher mortality rates in 2000–2003 and lower rates in more recent years. Adjustments should account for mortality rates declining more steeply than currently assumed. The decrease in mortality rates observed in the ART-CC occurred earlier in some countries than others, possibly because of earlier introduction of guidelines and policies for treating HIV [22]. Another explanation could be variation in the demographic characteristics of PLHIV across the countries [8]. Mortality rate decreases observed in ART-CC were not linear, for example, the decrease for Switzerland appeared to level off over time and so may have reached a steady state with little room for further improvement in the survival of PLHIV. The increase in the proportion of deaths in the ART-CC coded as unknown/unclassifiable in 2012–2015 compared with earlier years could be because of various reasons, one being that for some cohorts these later deaths had not been linked to cause of death information at the time of the data update.

### Comparison with literature

A European multicohort study by the D:A:D collaboration, containing some of the same cohorts as ART-CC, found the percentage of deaths because of AIDS to be 29% between 1999 and 2011 [2]. This is lower than the percentage found in the European ART-CC cohorts (43% in 2000–2003 dropping to 31% in 2008–2011), but not too dissimilar and could be explained by differences between the cohorts analysed. A Public Health England study found the percentage of deaths because of AIDS to be 58% between 1997 and 2012 [23], although this study also included PLHIV not on ART, possibly explaining the higher percentage. Studies in other high-income countries also found similar percentage of deaths because of AIDS, such as 40% in the Australian AHOD cohort between 1999 and 2004 [24], similar to the ART-CC's 43% for 2000–2003. The BC-CfE study in Vancouver, Canada, found the percentage of deaths because of AIDS to be 73% in 2005, falling to 20% in 2013 [25]. The 73% is much higher than the ART-CC's comparative 34% for 2004–2007, possibly because of different patient mix and guidelines. However, BC-CfE's 20% figure is almost identical to the ART-CC's 19% seen between 2012 and 2015. There is considerable evidence that, as shown in this study, mortality is highest during the first year of ART and decreases afterwards [5–7].

### Strengths and limitations

The HIV cohorts included in this analysis are a good representation of the PLHIV on ART in their countries in terms of patient mix and because of large sample sizes [10]. The under-ascertainment of deaths in the ART-CC because of LTFU may be a limitation, but registry linkages of deaths have been done in most cohorts. A French study from 2000 identified a large underreporting of deaths in FHDH itself, 62%, which dropped to 8% once other sources of mortality were accounted for [26]. A previous ART-CC analysis from 2012 gives self-reported completeness of death ascertainment by cohort and found that cohorts with lower completeness also had lower reported mortality rates [27]. LTFU in the ART-CC could be because of PLHIV becoming sicker and not turning up to appointments, conversely, it could be because of PLHIV being healthy enough to move to another clinic, cohort, or country [28]. Despite the percentage of AIDS-related deaths in the ART-CC being lower than in Spectrum, the cause of death-coding system in the ART-CC may over-estimate AIDS-related deaths because of the use of indirect evidence of AIDS, such as low CD4^+^ counts within the year before death [29]. Additionally, how excess mortality is defined can make comparisons of mortality rates between the ART-CC and Spectrum difficult. Differences between Spectrum and the ART-CC could be methodological or data-driven, which is also hard to ascertain. For comparability with Spectrum, important variables were not adjusted for, so assumptions were made that the characteristics of PLHIV in the ART-CC and Spectrum were similar, which may not be the case. For other analyses, some variables, such as geographical origin, hepatitis C status, and sociodemographic indicators, were not adjusted for as they were unavailable for many PLHIV.

### Implications

The findings of this study indicate that the assumptions around all-cause mortality for PLHIV on ART in high-income European settings should be adjusted in Spectrum. Mortality rates for PLHIV on ART were higher in 2000–2003 and declined more quickly than captured by Spectrum. Although when calculating excess mortality in the ART-CC, the mortality rates are broadly similar to those in Spectrum, the percentage of deaths because of AIDS among PLHIV in the ART-CC is much lower. These lower percentages seen in the ART-CC have also been recorded in other studies in high-income countries [2,24,25]. With increasingly effective ART, much of the excess mortality among PLHIV is because of other factors, such as higher levels of smoking, IDU, inflammatory markers, and hepatitis co-infection than in the general population [19–21]. This possibly indicates excess mortality in Spectrum should be broken into two categories: AIDS-related excess mortality, and non-AIDS-related excess mortality. Additionally, this study gives further information about the effect of duration of ART on mortality rates and cause-specific mortality among PLHIV on ART in high-income European countries. These findings are likely generalizable to other high-income settings. The new rates reported here in Supplementary Table 1 (model 1) have been incorporated into Spectrum for the 2019 round of UNAIDS HIV estimates.

## Acknowledgements

We thank all PLHIV, doctors, and study nurses associated with the participating cohort studies.

ART-CC cohorts included: AHIVCOS, AQUITAINE, ATHENA, Co-RIS, DHCS, FHDH, ICONA, PISCIS, SHCS, VACH.

Declaration of interests: The following members of the writing committee, or their institution, received fees from the following entities for work unrelated to this article. M.J.G. has received personal fees as an ad-hoc member of the Canadian HIV advisory boards of Gilead, Merck, and ViiV. The European Centre for Disease Prevention and Control funded Stichting HIV Monitoring for work by A.V.S. All other members of the writing committee declare no competing interests. S.A. has received travel grant from Gilead and ViiV and is member of Janssen-Cilag HIV board. J.B. has received research grants from AbbVie, Gilead, Merck, and ViiV; as well as personal fees from AbbVie, Gilead, Janssen, Merck, and ViiV. F.B. has served as a speaker for BMS, Gilead, MSD, and ViiV Healthcare, a consultant for Pierre Fabre and ViiV Healthcare, and has received research funding from Gilead and Janssen.

Funding: This work was supported by the UK Medical Research Council (MRC; grant number MR/J002380/1) and the UK Department for International Development (DFID) under the MRC/DFID Concordat agreement and is also part of the EDCTP2 programme supported by the European Union. The ART-CC is funded by the US National Institute on Alcohol Abuse and Alcoholism (U01-AA026209). J.A.C.S. is funded by National Institute for Health Research Senior Investigator award NF-SI-0611-10168. Data from 11 European cohorts were pooled within COHERE in EuroCoord. COHERE receives funding from the European Union Seventh Framework Programme (FP7/2007–2013) under EuroCoord grant agreement number 260694. Sources of funding of individual cohorts include the Agence Nationale de Recherche sur le SIDA et les hépatites virales (ANRS), the Institut National de la Santé et de la Recherche Médicale (INSERM), the French, Italian, and Spanish Ministries of Health, the Swiss National Science Foundation (grant 33CS30_134277), the Ministry of Science and Innovation and the Spanish Network for AIDS Research (RIS; ISCIII-RETIC RD06/006), the Stichting HIV Monitoring, the European Commission (EuroCoord grant 260694), and unrestricted grants from Abbott, Gilead, Tibotec-Upjohn, ViiV Healthcare, MSD, GlaxoSmithKline, Pfizer, Bristol-Myers Squibb, Roche, and Boehringer Ingelheim. The Danish HIV Cohort Study is funded by the Preben and Anne Simonsens Foundation.

### Conflicts of interest

There are no conflicts of interest.

## Supplementary Material

Supplemental Digital Content

## References

[1] Antiretroviral Therapy Cohort Collaboration. Survival of HIV-positive patients starting antiretroviral therapy between 1996 and 2013: a collaborative analysis of cohort studies. *Lancet HIV* 2017; 4:e349–e356. 28501495 10.1016/S2352-3018(17)30066-8 PMC5555438

[2] Smith CJ Ryom L Weber R Morlat P Pradier C Reiss P D:A:D Study Group. Trends in underlying causes of death in people with HIV from 1999 to 2011 (D:A:D): a multicohort collaboration. *Lancet* 2014; 384:241–248. 25042234 10.1016/S0140-6736(14)60604-8

[3] Iacob SA Iacob DG Jugulete G Improving the adherence to antiretroviral therapy, a difficult but essential task for a successful HIV treatment-clinical points of view and practical considerations. *Front Pharmacol* 2017; 8:831. 29218008 10.3389/fphar.2017.00831 PMC5703840

[4] Eholie SP Badje A Kouame GM N’takpe JB Moh R Danel C Antiretroviral treatment regardless of CD4 count: the universal answer to a contextual question. *AIDS Res Ther* 2016; 13:27. 27462361 10.1186/s12981-016-0111-1 PMC4960900

[5] Young J Psichogiou M Meyer L Ayayi S Grabar S Opportunistic Infections Project Team of COHERE. CD4 cell count and the risk of AIDS or death in HIV-Infected adults on combination antiretroviral therapy with a suppressed viral load: a longitudinal cohort study from COHERE. *PLoS Med* 2012; 9:e1001194. 22448150 10.1371/journal.pmed.1001194 PMC3308938

[6] Ren L Li J Zhou S Xia X Xie Z Liu P Prognosis of HIV patients receiving antiretroviral therapy according to CD4 counts: a long-term follow-up study in Yunnan, China. *Sci Rep* 2017; 7:9595. 28852017 10.1038/s41598-017-10105-7 PMC5575268

[7] Walker AS Prendergast AJ Mugyenyi P Munderi P Hakim J Kekitiinwa A DART and ARROW trial teams. Mortality in the year following antiretroviral therapy initiation in HIV-infected adults and children in Uganda and Zimbabwe. *Clin Infect Dis* 2012; 55:1707–1718. 22972859 10.1093/cid/cis797 PMC3501336

[8] Nakagawa F Phillips AN Lundgren JD Update on HIV in Western Europe. *Curr HIV/AIDS Rep* 2014; 11:177–185. 24659343 10.1007/s11904-014-0198-8 PMC4032460

[9] Stover J Andreev K Slaymaker E Gopalappa C Sabin K Velasquez C Updates to the spectrum model to estimate key HIV indicators for adults and children. *AIDS* 2014; 28: Suppl 4: S427–S434. 25406748 10.1097/QAD.0000000000000483 PMC4247263

[10] May MT Ingle SM Costagliola D Justice AC de Wolf F Cavassini M Antiretroviral Cohort Collaboration. Cohort Profile: Antiretroviral Therapy Cohort Collaboration (ART-CC). *Int J Epidemiol* 2014; 43:691–702. 23599235 10.1093/ije/dyt010 PMC4052127

[11] Gange SJ Kitahata MM Saag MS Bangsberg DR Bosch RJ Brooks JT Cohort profile: The North American AIDS Cohort Collaboration on Research and Design (NA-ACCORD). *Int J Epidemiol* 2007; 36:294–301. 17213214 10.1093/ije/dyl286 PMC2820873

[12] Busch M Comparing Austrian HIV Cohort Study with the monitoring of drug related infectious diseases. In: EU expert meeting on the EMCDDA key epidemiological indicator Drug Related Infectious Diseases (DRID). Lisbon; 2012.

[13] Kowalska JD Friis-Moller N Kirk O Bannister W Mocroft A Sabin C CoDe Working Group; D:A:D Study Group. The Coding Causes of Death in HIV (CoDe) Project: initial results and evaluation of methodology. *Epidemiology* 2011; 22:516–523. 21522013 10.1097/EDE.0b013e31821b5332

[14] Yiannoutsos CT Johnson LF Boulle A Musick BS Gsponer T Balestre E International Epidemiologic Databases to Evaluate AIDS (IeDEA) Collaboration. Estimated mortality of adult HIV-infected patients starting treatment with combination antiretroviral therapy. *Sex Transm Infect* 2012; 88: Suppl 2: i33–i43. 23172344 10.1136/sextrans-2012-050658 PMC3512431

[15] UNAIDS. UNAIDS 2018 data. 2018.

[16] Royston P Parmar MK Flexible parametric proportional-hazards and proportional-odds models for censored survival data, with application to prognostic modelling and estimation of treatment effects. *Stat Med* 2002; 21:2175–2197. 12210632 10.1002/sim.1203

[17] Alejos B Hernando V Iribarren J Gonzalez-Garcia J Hernando A Santos J CoRIS (Cohort of the Spanish Network on HIVAIDS Research). Overall and cause-specific excess mortality in HIV-positive persons compared with the general population: role of HCV coinfection. *Medicine (Baltimore)* 2016; 95:e4727. 27603368 10.1097/MD.0000000000004727 PMC5023891

[18] Aldaz P Moreno-Iribas C Egues N Irisarri F Floristan Y Sola-Boneta J Mortality by causes in HIV-infected adults: comparison with the general population. *BMC Public Health* 2011; 11:300. 21569323 10.1186/1471-2458-11-300 PMC3112125

[19] Platt L Easterbrook P Gower E McDonald B Sabin K McGowan C Prevalence and burden of HCV co-infection in people living with HIV: a global systematic review and meta-analysis. *Lancet Infect Dis* 2016; 16:797–808. 26922272 10.1016/S1473-3099(15)00485-5

[20] Reynolds NR Cigarette smoking and HIV: more evidence for action. *AIDS Educ Prev* 2009; 21: 3 Suppl: 106–121. 19537958 10.1521/aeap.2009.21.3_supp.106 PMC3248054

[21] Degenhardt L Peacock A Colledge S Leung J Grebely J Vickerman P Global prevalence of injecting drug use and sociodemographic characteristics and prevalence of HIV, HBV, and HCV in people who inject drugs: a multistage systematic review. *Lancet Glob Health* 2017; 5:e1192–e1207. 29074409 10.1016/S2214-109X(17)30375-3 PMC5683738

[22] HIV in Europe. National HIV Testing Guidelines. 2018.

[23] Croxford S Kitching A Desai S Kall M Edelstein M Skingsley A Mortality and causes of death in people diagnosed with HIV in the era of highly active antiretroviral therapy compared with the general population: an analysis of a national observational cohort. *Lancet Public Health* 2017; 2:e35–e46. 29249478 10.1016/S2468-2667(16)30020-2

[24] Petoumenos K Law MG Australian HIV Observational Database. Risk factors and causes of death in the Australian HIV Observational Database. *Sex Health* 2006; 3:103–112. 16800396 10.1071/sh05045

[25] Lima VD Lourenco L Yip B Hogg RS Phillips P Montaner JSG AIDS incidence and AIDS-related mortality in British Columbia, Canada, between 1981 and 2013: a retrospective study. *Lancet HIV* 2015; 2:e92–e97. 25780802 10.1016/S2352-3018(15)00017-X PMC4357843

[26] Lewden C Jougla E Alioum A Pavillon G Lievre L Morlat P Number of deaths among HIV-infected adults in France in 2000, three-source capture-recapture estimation. *Epidemiol Infect* 2006; 134:1345–1352. 16690003 10.1017/S095026880600639X PMC2870522

[27] May MT Hogg RS Justice AC Shepherd BE Costagliola D Ledergerber B Antiretroviral Therapy Cohort Collaboration (ART-CC). Heterogeneity in outcomes of treated HIV-positive patients in Europe and North America: relation with patient and cohort characteristics. *Int J Epidemiol* 2012; 41:1807–1820. 23148105 10.1093/ije/dys164 PMC3535877

[28] Trickey A May MT Vehreschild J Obel N Gill MJ Crane H Cause-specific mortality in HIV-positive patients who survived ten years after starting antiretroviral therapy. *PLoS One* 2016; 11:e0160460. 27525413 10.1371/journal.pone.0160460 PMC4985160

[29] Ingle SM May MT Gill MJ Mugavero MJ Lewden C Abgrall S Antiretroviral Therapy Cohort Collaboration. Impact of risk factors for specific causes of death in the first and subsequent years of antiretroviral therapy among HIV-infected patients. *Clin Infect Dis* 2014; 59:287–297. 24771333 10.1093/cid/ciu261 PMC4073781

