# Supplementary material for: Parameter estimates for trends and patterns of excess mortality among persons on antiretroviral therapy in high-income European settings
Source: AIDS. 2019 Nov 11;33(Suppl 3):S271–81. doi: 10.1097/QAD.0000000000002387 (PMC6919232; doi:10.1097/QAD.0000000000002387)
Supplement: Supplemental Digital Content [file aids-33-s271-s001.docx]

**Parameter estimates for trends and patterns of excess mortality among persons on ART in high-income European settings**

**Supplementary table 1:** Adjusted incidence rate ratios (95% confidence intervals) for mortality, and the baseline mortality rates for models with a) 0-6 months, 7-12 months, 1+ years as the duration of ART; and b) 0-1, 1-2, 2-3, 3-4, 4+ years as the duration on ART

|  | **Adjusted incidence rate ratios (95% confidence interval)** | |
| --- | --- | --- |
| **Duration on ART** | **Model 1** | **Model 2** |
| 0-6 months | 1 | NA |
| 7-12 months | 0.52 (0.47, 0.59) | NA |
| 1+ year | 0.35 (0.33, 0.38) | NA |
|  |  |  |
| 0-1 year | NA | 1 |
| 1-2 years | NA | 0.52 (0.47, 0.57) |
| 2-3 years | NA | 0.49 (0.44, 0.54) |
| 3-4 years | NA | 0.43 (0.38, 0.47) |
| 4+ years | NA | 0.45 (0.42, 0.48) |
|  |  |  |
| Calendar year |  |  |
| 2000-2003 | 1.82 (1.69, 1.96) | 1.78 (1.65, 1.93) |
| 2004-2007 | 1.38 (1.29, 1.47) | 1.37 (1.28, 1.47) |
| 2008-2011 | 1 | 1 |
| 2012-2015 | 0.65 (0.59, 0.71) | 0.65 (0.59, 0.71) |
|  |  |  |
| Male | 1 | 1 |
| Female | 0.81 (0.76, 0.87) | 0.81 (0.76, 0.87) |
|  |  |  |
| Age |  |  |
| 16-24 years | 1 | 1 |
| 25-34 years | 1.24 (0.92, 1.67) | 1.23 (0.92, 1.66) |
| 35-44 years | 1.83 (1.36, 2.44) | 1.83 (1.37, 2.45) |
| 45+ years | 3.68 (2.75, 4.92) | 3.71 (2.78, 4.96) |
|  |  |  |
| Non-IDU transmission | 1 | 1 |
| IDU transmission | 2.62 (2.45, 2.80) | 2.63 (2.46, 2.81) |
|  |  |  |
| Baseline CD4 count cells/μL |  |  |
| 0-49 | 1 | 1 |
| 50-99 | 0.85 (0.78, 0.93) | 0.85 (0.78, 0.92) |
| 100-199 | 0.63 (0.58, 0.68) | 0.63 (0.58, 0.68) |
| 200-249 | 0.50 (0.46, 0.56) | 0.50 (0.45, 0.55) |
| 250-349 | 0.44 (0.40, 0.48) | 0.43 (0.40, 0.47) |
| 350-499 | 0.37 (0.34, 0.41) | 0.37 (0.34, 0.41) |
| 500+ | 0.41 (0.36, 0.45) | 0.41 (0.36, 0.45) |
|  |  |  |
| Baseline mortality rate | 0.0147 | 0.0112 |

**Supplementary table 2:** Country-specific mortality rates (with 95% confidence intervals for ART-CC estimates) for all-cause and AIDS-related mortality

|  |  | **All-cause mortality rate** | | **AIDS-related mortality rate** | |  |
| --- | --- | --- | --- | --- | --- | --- |
| **Country** | **Years** | **Spectrum** | **ART-CC** | **Spectrum** | **ART-CC, cause-specific¶** | **ART-CC, excess†** |
| Austria | 2000-2003 | 0.0130 | 0.0228 (0.0162, 0.0295) | 0.0098 | 0.0064 (0.0030, 0.0098) | 0.0189 (0.0126, 0.0253) |
|  | 2004-2007 | 0.0107 | 0.0210 (0.0162, 0.0258) | 0.0074 | 0.0068 (0.0042, 0.0095) | 0.0154 (0.0109, 0.0199) |
|  | 2008-2011 | 0.0087 | 0.0147 (0.0117, 0.0178) | 0.0052 | 0.0037 (0.0022, 0.0052) | 0.0096 (0.0068, 0.0125) |
|  | 2012-2015 | 0.0063 | 0.0100 (0.0076, 0.0124) | 0.0028 | 0.0012 (0.0004, 0.0020) | 0.0056 (0.0034, 0.0077) |
| Denmark | 2000-2003 | 0.0127 | 0.0227 (0.0174, 0.0279) | 0.0092 | 0.0103 (0.0068, 0.0138) | 0.0167 (0.0117, 0.0217) |
|  | 2004-2007 | 0.0114 | 0.0208 (0.0166, 0.0249) | 0.0074 | 0.0042 (0.0024, 0.0061) | 0.0142 (0.0103, 0.0181) |
|  | 2008-2011 | 0.0099 | 0.0080 (0.0058, 0.0101) | 0.0055 | 0.0012 (0.0004, 0.0021) | 0.0026 (0.0008, 0.0044) |
|  | 2012-2015 | 0.0078 | 0.0060 (0.0039, 0.0081) | 0.0031 | 0.0000 (0.0000, 0.0000) | 0.0012 (0.0000, 0.0027) |
| France | 2000-2003 | 0.0099 | 0.0128 (0.0117, 0.0138) | 0.0072 | 0.0048 (0.0042, 0.0054) | 0.0089 (0.0079, 0.0099) |
|  | 2004-2007 | 0.0090 | 0.0082 (0.0075, 0.0088) | 0.0060 | 0.0018 (0.0015, 0.0021) | 0.0042 (0.0036, 0.0048) |
|  | 2008-2011 | 0.0078 | 0.0060 (0.0055, 0.0066) | 0.0045 | 0.0013 (0.0011, 0.0016) | 0.0021 (0.0017, 0.0026) |
|  | 2012-2015 | 0.0064 | 0.0017 (0.0013, 0.0021) | 0.0028 | 0.0003 (0.0001, 0.0004) | 0.0000 (0.0000, 0.0000) |
| Italy | 2000-2003 | 0.0163 | 0.0178 (0.0136, 0.0220) | 0.0122 | 0.0091 (0.0062, 0.0121) | 0.0150 (0.0109, 0.0192) |
|  | 2004-2007 | 0.0140 | 0.0134 (0.0097, 0.0170) | 0.0095 | 0.0044 (0.0023, 0.0064) | 0.0111 (0.0075, 0.0147) |
|  | 2008-2011 | 0.0118 | 0.0080 (0.0055, 0.0106) | 0.0068 | 0.0026 (0.0012, 0.0040) | 0.0050 (0.0026, 0.0074) |
|  | 2012-2015 | 0.0099 | 0.0063 (0.0044, 0.0082) | 0.0042 | 0.0016 (0.0007, 0.0026) | 0.0034 (0.0017, 0.0051) |
| Netherlands | 2000-2003 | 0.0112 | 0.0107 (0.0089, 0.0126) | 0.0084 | 0.0035 (0.0025, 0.0045) | 0.0070 (0.0053, 0.0087) |
|  | 2004-2007 | 0.0096 | 0.0115 (0.0100, 0.0129) | 0.0067 | 0.0038 (0.0029, 0.0046) | 0.0078 (0.0065, 0.0092) |
|  | 2008-2011 | 0.0076 | 0.0099 (0.0088, 0.0110) | 0.0046 | 0.0019 (0.0014, 0.0024) | 0.0054 (0.0044, 0.0064) |
|  | 2012-2015 | 0.0058 | 0.0076 (0.0066, 0.0085) | 0.0026 | 0.0006 (0.0003, 0.0008) | 0.0029 (0.0021, 0.0037) |
| Spain | 2000-2003 | 0.0129 | 0.0166 (0.0147, 0.0185) | 0.0100 | 0.0043 (0.0034, 0.0053) | 0.0131 (0.0113, 0.0149) |
|  | 2004-2007 | 0.0108 | 0.0149 (0.0136, 0.0163) | 0.0077 | 0.0040 (0.0033, 0.0047) | 0.0110 (0.0097, 0.0123) |
|  | 2008-2011 | 0.0089 | 0.0107 (0.0098, 0.0116) | 0.0055 | 0.0033 (0.0028, 0.0038) | 0.0071 (0.0062, 0.0079) |
|  | 2012-2015 | 0.0071 | 0.0071 (0.0060, 0.0081) | 0.0033 | 0.0013 (0.0008, 0.0017) | 0.0035 (0.0026, 0.0044) |
| Switzerland | 2000-2003 | 0.0136 | 0.0161 (0.0130, 0.0191) | 0.0107 | 0.0052 (0.0034, 0.0070) | 0.0128 (0.0098, 0.0158) |
|  | 2004-2007 | 0.0115 | 0.0088 (0.0070, 0.0105) | 0.0083 | 0.0023 (0.0014, 0.0032) | 0.0051 (0.0035, 0.0067) |
|  | 2008-2011 | 0.0090 | 0.0066 (0.0053, 0.0079) | 0.0057 | 0.0011 (0.0005, 0.0016) | 0.0030 (0.0019, 0.0042) |
|  | 2012-2015 | 0.0063 | 0.0058 (0.0041, 0.0074) | 0.0030 | 0.0001 (0.0000, 0.0004) | 0.0019 (0.0005, 0.0032) |
| **All** | **2000-2003** | **0.0121** | **0.0151 (0.0130, 0.0171)** | **0.0091** | **0.0049 (0.0044, 0.0053)** | **0.0115 (0.0095, 0.0135)** |
|  | **2004-2007** | **0.0115** | **0.0118 (0.0101, 0.0134)** | **0.0074** | **0.0028 (0.0026, 0.0031)** | **0.0082 (0.0066, 0.0098)** |
|  | **2008-2011** | **0.0097** | **0.0082 (0.0070, 0.0094)** | **0.0054** | **0.0020 (0.0018, 0.0022)** | **0.0045 (0.0034, 0.0056)** |
|  | **2012-2015** | **0.0078** | **0.0049 (0.0039, 0.0060)** | **0.0032** | **0.0007 (0.0005, 0.0008)** | **0.0021 (0.0013, 0.0029)** |

**¶** Calculated using cause-specific death coding

**†** Calculated as the excess mortality of PLHIV above that of the general population

**Supplementary table 3:** European mortality rates (percentage of 2000-03 value) by calendar year group for each CD4 cell count category at the start of ART

|  | **CD4 count (cells/μL) at start of ART** | | | | | | |
| --- | --- | --- | --- | --- | --- | --- | --- |
| **Year group** | **0-49** | **50-99** | **100-199** | **200-249** | **250-349** | **350-499** | **500+** |
| **2000-03** | 0.0963 (NA) | 0.0252 (NA) | 0.0157 (NA) | 0.0060 (NA) | 0.0040 (NA) | 0.0023 (NA) | 0.0034 (NA) |
| **2004-07** | 0.0710 (74%) | 0.0192 (76%) | 0.0105 (67%) | 0.0040 (67%) | 0.0037 (93%) | 0.0020 (87%) | 0.0036 (106%) |
| **2008-11** | 0.0514 (53%) | 0.0153 (61%) | 0.0079 (50%) | 0.0030 (50%) | 0.0024 (60%) | 0.0017 (74%) | 0.0026 (76%) |
| **2012-15** | 0.0332 (34%) | 0.0090 (36%) | 0.0056 (36%) | 0.0022 (37%) | 0.0015 (38%) | 0.0010 (43%) | 0.0017 (50%) |

**Supplementary table 4:** Distribution of AIDS causes of death in the ART-CC for differing assumptions around unknown/unclassifiable causes of deaths

|  | **Percentage of deaths due to AIDS** | | | |
| --- | --- | --- | --- | --- |
| **Assumption** | **2000-2003** | **2004-2007** | **2008-2011** | **2012-2015** |
| None of the unknown/unclassifiable deaths were due to AIDS (low estimate) | 496 (36%) | 454 (27%) | 429 (25%) | 90 (13%) |
| Assuming all of the unknown/unclassifiable deaths were due to AIDS (high estimate) | 721 (52%) | 785 (46%) | 767 (44%) | 364 (51%) |
| Some of the unknown/unclassifiable deaths were due to AIDS, proportionate to the actual numbers of AIDS deaths (middle estimate) | 606 (44%) | 561 (33%) | 533 (31%) | 149 (21%) |

**Supplementary table 5:** Country-specific percentages of deaths (confidence bounds*) due to AIDS among people living with HIV in Spectrum and the ART-CC, calculated as cause-specific and excess mortality

|  |  | **Percentage of deaths due to AIDS among PLHIV** | | |
| --- | --- | --- | --- | --- |
| **Country** | **Years** | **Spectrum** | **ART-CC, cause-specific¶** | **ART-CC, excess†** |
| Austria | 2000-2003 | 75.3% | 29.8% (29.8%, 31.9%) | 83.0% (51.1%, 100%) |
|  | 2004-2007 | 68.6% | 33.8% (33.8%, 35.1%) | 73.3% (49.2%, 100%) |
|  | 2008-2011 | 58.9% | 29.4% (26.1%, 38.0%) | 65.6% (44.2%, 92.6%) |
|  | 2012-2015 | 43.2% | 17.1% (12.9%, 37.1%) | 55.9% (33.0%, 85.5%) |
| Denmark | 2000-2003 | 72.4% | 52.0% (46.7%, 58.7%) | 73.6% (48.7%, 100.0%) |
|  | 2004-2007 | 64.7% | 23.8% (20.8%, 39.6%) | 68.5% (47.4%, 94.9%) |
|  | 2008-2011 | 55.3% | 32.7% (15.4%, 53.9%) | 33.0% (10.3%, 61.1%) |
|  | 2012-2015 | 39.7% | 0.0% (0.0%, 100.0%) | 19.9% (0.0%, 51.3%) |
| France | 2000-2003 | 72.7% | 50.8% (38.5%, 63.5%) | 69.4% (60.2%, 79.6%) |
|  | 2004-2007 | 66.5% | 36.7% (22.5%, 58.6%) | 51.4% (43.6%, 59.9%) |
|  | 2008-2011 | 57.6% | 31.5% (22.2%, 49.4%) | 35.1% (27.7%, 43.2%) |
|  | 2012-2015 | 43.0% | 31.1% (17.6%, 60.8%) | 0.0% (0.0%, 0.0%) |
| Italy | 2000-2003 | 74.5% | 54.2% (54.2%, 55.6%) | 84.4% (57.6%, 100.0%) |
|  | 2004-2007 | 68.0% | 35.9% (34.0%, 41.5%) | 83.0% (52.8%, 100.0%) |
|  | 2008-2011 | 57.9% | 41.0% (33.3%, 46.2%) | 62.4% (31.3%, 100.0%) |
|  | 2012-2015 | 42.1% | 34.1% (27.3%, 45.5%) | 53.5% (25.1%, 91.8%) |
| Netherlands | 2000-2003 | 75.2% | 41.8% (35.1%, 48.5%) | 65.4% (47.4%, 87.2%) |
|  | 2004-2007 | 69.2% | 38.2% (34.6%, 46.8%) | 68.5% (54.5%, 84.6%) |
|  | 2008-2011 | 59.8% | 29.4% (20.0%, 50.9%) | 54.3% (43.3%, 66.6%) |
|  | 2012-2015 | 43.6% | 21.4% (7.8%, 72.4%) | 38.6% (27.5%, 50.7%) |
| Spain | 2000-2003 | 77.1% | 31.1% (27.6%, 35.3%) | 78.8% (65.5%, 94.3%) |
|  | 2004-2007 | 70.8% | 30.2% (27.9%, 35.1%) | 73.6% (63.3%, 85.0%) |
|  | 2008-2011 | 61.7% | 33.9% (32.1%, 37.9%) | 66.4% (57.2%, 76.6%) |
|  | 2012-2015 | 45.8% | 20.3% (18.7%, 24.1%) | 49.0% (35.1%, 64.5%) |
| Switzerland | 2000-2003 | 78.2% | 34.5% (31.0%, 42.5%) | 79.6% (57.7%, 100.0%) |
|  | 2004-2007 | 71.8% | 29.6% (25.5%, 36.7%) | 58.0% (38.4%, 82.8%) |
|  | 2008-2011 | 62.5% | 18.2% (16.2%, 32.3%) | 46.4% (28.2%, 68.4%) |
|  | 2012-2015 | 46.3% | 2.1% (2.1%, 23.4%) | 32.1% (8.9%, 60.9%) |
| **All** | **2000-2003** | **74.7%** | **45.3% (38.4%, 52.9%)** | **75.3% (60.3%, 95.2%)** |
|  | **2004-2007** | **68.4%** | **34.3% (27.3%, 46.4%)** | **66.4% (53.7%, 88.2%)** |
|  | **2008-2011** | **59.1%** | **34.1% (27.5%, 44.7%)** | **52.4% (40.6%, 72.8%)** |
|  | **2012-2015** | **43.6%** | **26.7% (19.0%, 46.0%)** | **30.7% (25.5%, 63.7%)** |

**¶** Calculated using cause-specific death coding

**†** Calculated as the excess mortality of PLHIV above that of the general population

*The cause-specific bounds are from making different assumptions about the unknown/unclassifiable mortality (see supplementary table 4), whilst the 95% confidence bounds for the excess mortality were calculated using a Bayesian framework
